# Author Correction: Phonemic segmentation of narrative speech in human cerebral cortex

**DOI:** 10.1038/s41467-023-42951-7

**Published:** 2023-11-01

**Authors:** Xue L. Gong, Alexander G. Huth, Fatma Deniz, Keith Johnson, Jack L. Gallant, Frédéric E. Theunissen

**Affiliations:** 1 Helen Wills Neuroscience Institute, University of California, Berkeley, Berkeley, 94720 CA USA; 2 Departments of Neuroscience and Computer Science, University of Texas, Austin, Austin, 78712 TX USA; 3 Faculty of Electrical Engineering and Computer Science, Technische Universität Berlin, Berlin, 10587 Berlin Germany; 4 Department of Linguistics, University of California, Berkeley, Berkeley, 94720 CA USA; 5 Department of Psychology, University of California, Berkeley, Berkeley, 94720 CA USA; 6 Department of Integrative Biology, University of California, Berkeley, Berkeley, 94720 CA USA

**Keywords:** Cortex, Language, Neural encoding

Correction to: *Nature Communications* 10.1038/s41467-023-39872-w, published online 18 July 2023

The original version of this Article contained an error in Fig. 6, in which the brain maps in panels b and d were inadvertently swapped. This has been corrected in both the PDF and HTML versions of the Article.

