# Supplementary material for: Phonemic segmentation of narrative speech in human cerebral cortex
Source: Nat Commun. 2023 Jul 18;14:4309. doi: 10.1038/s41467-023-39872-w (PMC10354060; doi:10.1038/s41467-023-39872-w)
Supplement: Supplementary file 1 — Supplementary Information [file 41467_2023_39872_MOESM1_ESM.pdf]

Phonemic segmentation of narrative speech in human cerebral cortex

Gong et al

1 Supplementary Figures

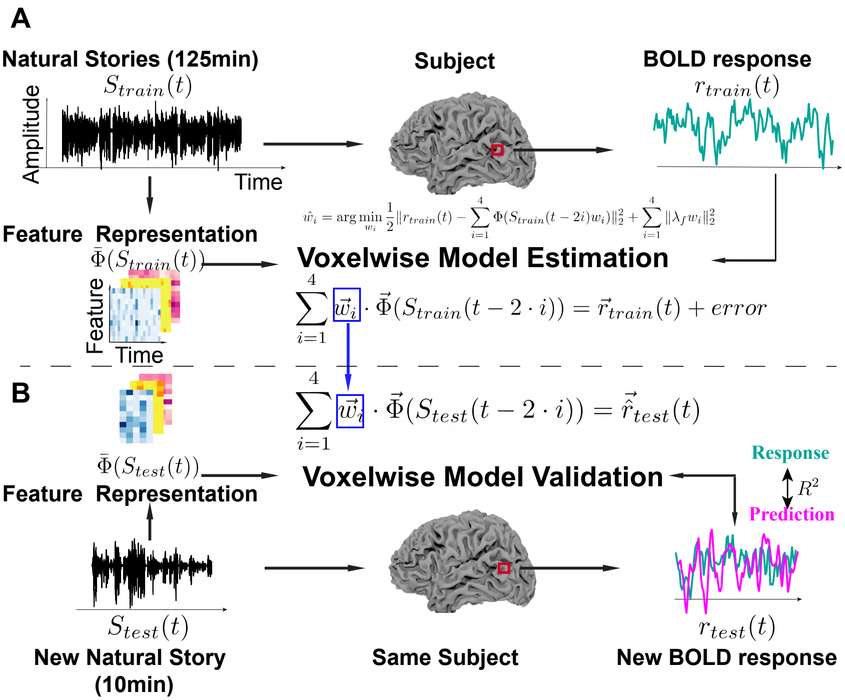

Supplementary Figure 1 Voxelwise Modeling.A. Voxelwise modeling (VM) estimation: Regularized linear regression was used to predict BOLD activity in individual voxels. Regression weights ($\bar{w}$) were estimated for each voxel and each feature space. The linear regression used the stimulus features in the four time windows preceding the BOLD activity at time t; the time windows are $2sec$ long, corresponding to the scanner TR (0.5Hz). B. **Voxelwise Model Validation:** The quality of the models' predictions was assessed using one $10-min$ story not included during model estimation (validation story). The BOLD (green) response to this validation story (green) was compared to the prediction (magenta) to calculate the cross-validated R^2.

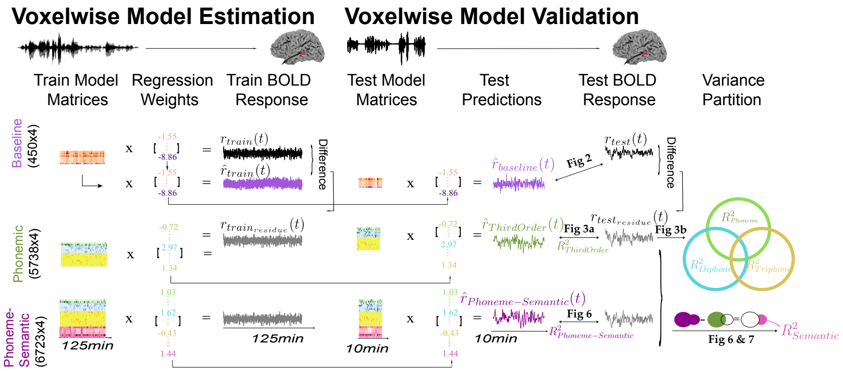

Supplementary Figure 2 Variance Partitioning. Variance partitioning was used to obtain the BOLD response uniquely explained by different phonemic and semantic features. Before fitting models that capture particular phonemes or semantic meanings, the fraction of the BOLD response predicted by the baseline model was subtracted, in order to eliminate the BOLD responses explained by the mere presence versus absence of auditory speech stimuli. We then fitted a joint phonemic model with all phonemic features (single phonemes, diphones and triphones) and a joint phonemic-semantic model with all the phonemic and semantic features. Afterwards, we used variance partitioning to obtain unique variance explained by each phonemic feature and all the possible combinations of these phonemic features (single phonemes + diphones, diphones + triphones, single phonemes + triphone, single phonemes + diphones + triphones). The unique variance explained by semantic feature is obtained by subtracting from the variance explained of phonemic-semantic model from that of phonemic models.

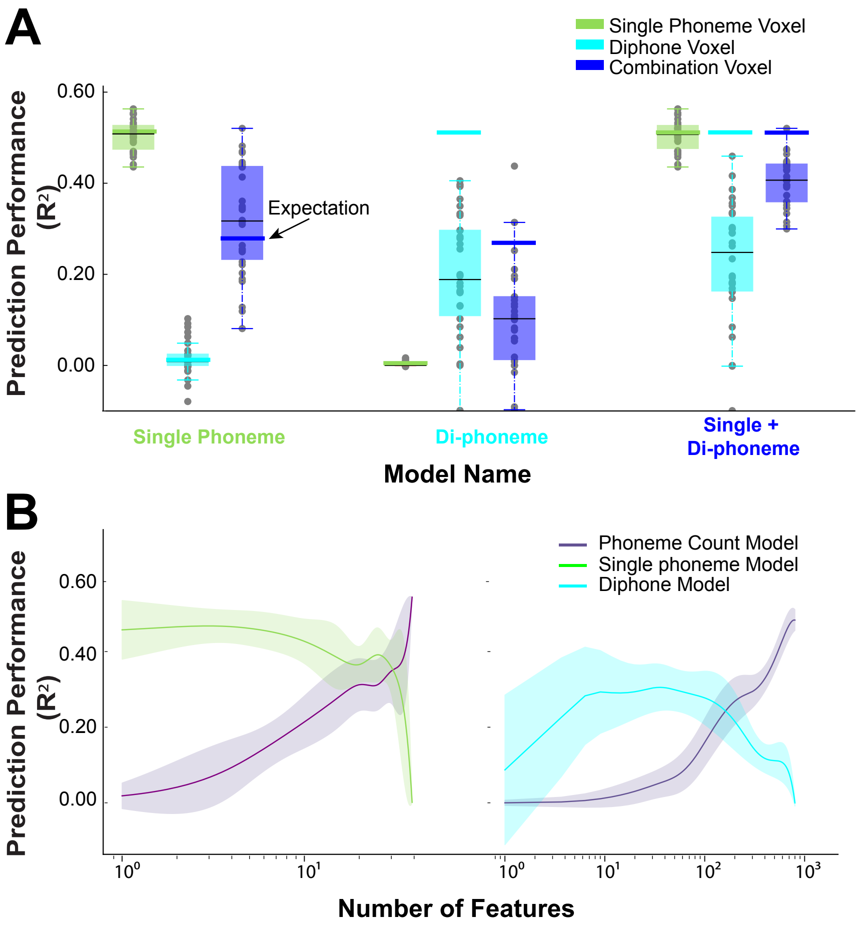

Supplementary Figure 3 Validation by simulations: effect of voxel sensitivity. Panel A. The coefficient of determination, R^2, was calculated for model voxels sensitive either to three single phonemes (green), three diphones (cyan) or the combination of three single and three single diphones (blue). For each voxel (n=1), we repeated this procedure 30 times (Y=30 independent experiments) to generate a distribution of simulated results. The three groups on the x-axis correspond to the VMs based on single phonemes features, diphone features or both. The solid bold colored lines (labeled as Expectation) show the ground truth. **Panel B.** The simulation for single phoneme (left panel) and diphone (right panel) voxels was repeated by systematically varying the rate of phonemic units (x-axis) with non-zero weights; from highly sensitive model voxels that respond 1 phonemic unit to less sensitive units that respond to 10s or 100s of phones and diphones. To assess the sensitivity of our method (given our recording time of 3737 TRs), we compared the prediction obtained from the correct phonemic identity VM (single phoneme VM, red line for left plot and diphone VM, green line for right plot) to the VM based solely on the phoneme count (purple line). Error bars are SEM obtained by repeating the simulation by using different random samples of phonemic units with non-zero weights.

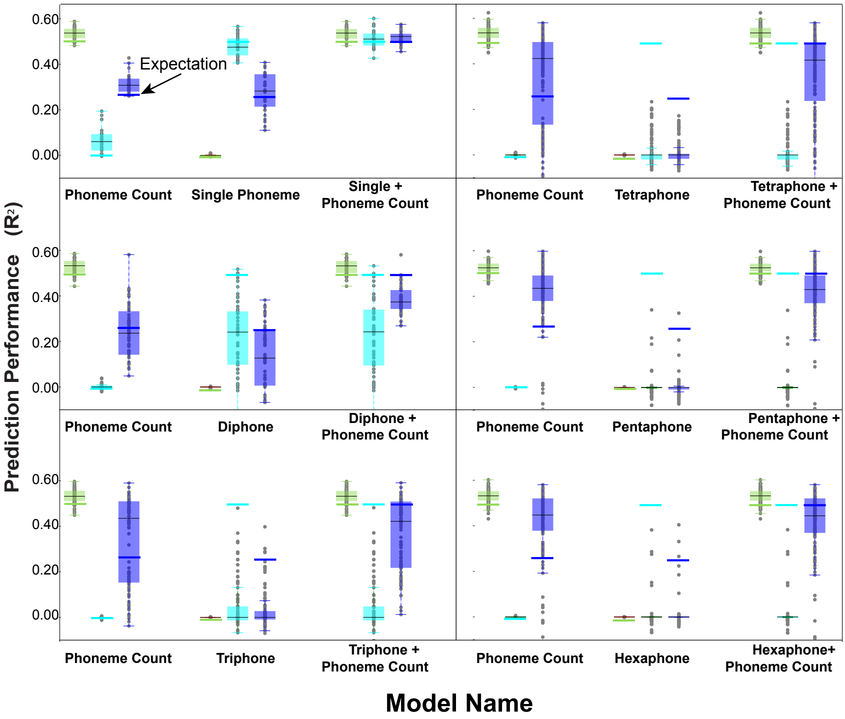

Supplementary Figure 4 Validation by simulation: sensitivity to longer combinations of phonemes. To explore the limits of phonemic combinations that could be recovered from our dataset, we also simulated responses to higher order combinations. In this figure (similarly to Figure Supplementary Figure 3A), each subpanel shows the results obtained applying nested VM to sets of three putative voxels: one sensitive to the phoneme count, one to different phonemic combinations (single phoneme, diphone, triphone, tetraphone, pentaphone, and hexaphone) and one to the mixture of both. For each voxel (n=1), we repeated this procedure 30 times for single phoneme simulation, 50 times for diphones, and 150 times for triphone, tetraphone, pentaphone and hexaphone based VMs to obtain the distribution of the prediction performance. As shown in the plot, given the measured SNR and about 2 hours of data, one cannot recover the signals of putative voxels sensitive for phonemic combinations beyond the triphone.

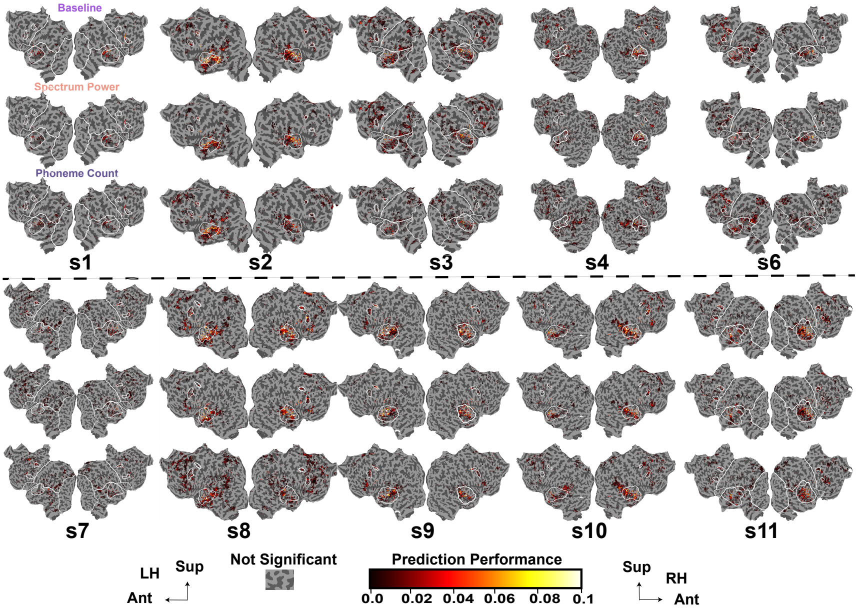

Supplementary Figure 5 Prediction performance of auditory processing on flat maps for the remaining 10 subjects. The cortical flatmaps on the first rows show the significant prediction performance of the Baseline VM model for all the subjects in the study (Subject 5 is shown on the main Figure 2). The Baseline VM uses the time varying power spectrum and the phoneme count as features. The second and third row show the additive contribution of the spectrum and phoneme count features to this auditory Baseline VM.

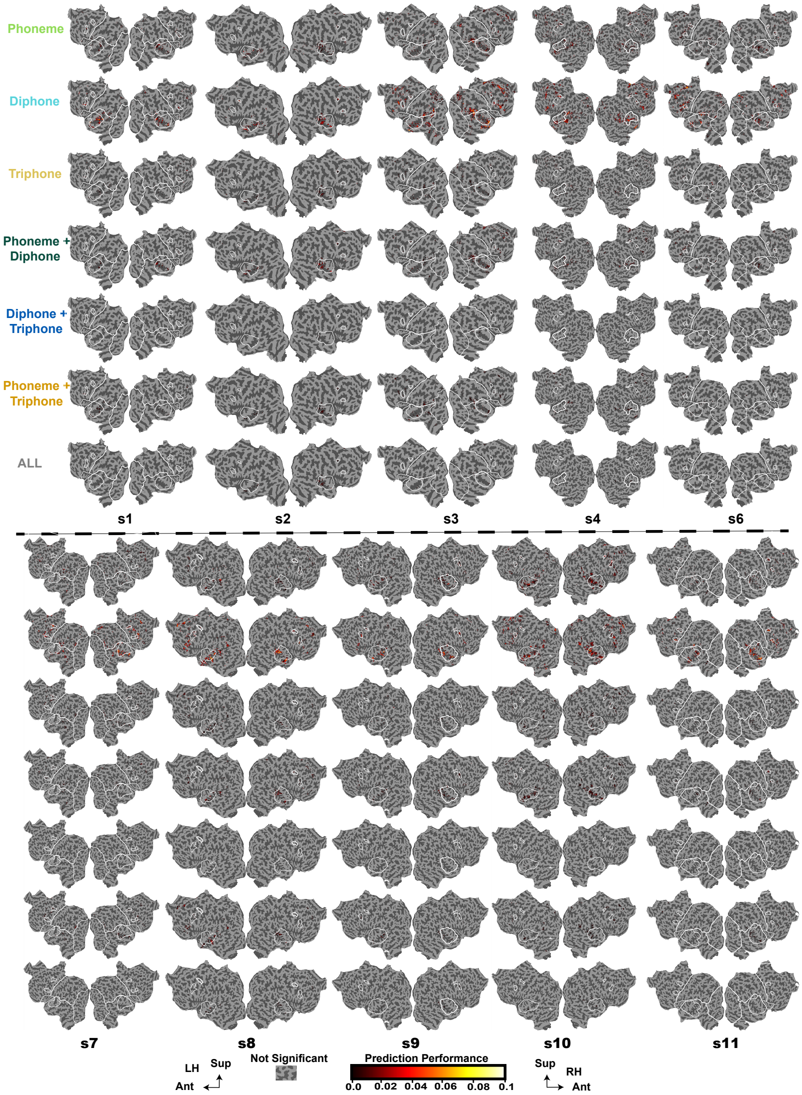

Supplementary Figure 6 Prediction performance of phonemic processing on flat maps for the remaining 10 **subjects.** Each column of the cortical flatmaps shows the significant prediction performance of the unique contributions from single phoneme, diphone, triphone, phoneme + diphone, diphone + triphone, phoneme + triphone and single phoneme + diphone + triphone features using variance partitioning for each subject. (Subject 5 is shown on the main Figure 3).

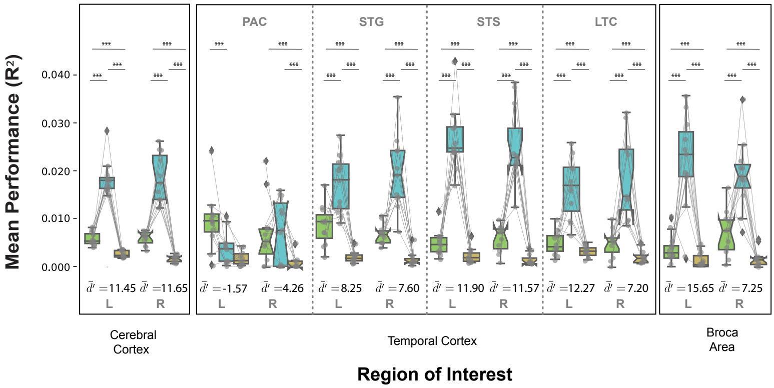

Supplementary Figure 7 Phonemic processing: hemispheric analysis. In order to examine whether the significant prediction performance from single phoneme, diphone and triphone features varied significantly between left (box) and right (notched box) hemispheres across regions of interest, we performed a linear mixed-effect model with post-hoc Tukey test. The statistics are derived from the performance of significant phonemic voxels for each ROI ($n = 0 - 1,973$ voxels) over 11 independent subjects. All tests are two sided and corrected for multiple comparisons. The exact p values can be found in the Laterality of the phonemic representation and segmentation section. The boxplots are defined the same way as in Figure 4 in the manuscript. The dots show the data of individual subjects with grey dots (*$p < 0.05$, **$p < 0.01$ and ***$p < 0.001$). In STG, STS and Broca's area, the diphone feature space explains significantly more variance than single phoneme or triphone features in the left hemisphere than that in the right hemisphere. It indicates that the diphone segmentation for phonemic processing is more prominent in the left hemisphere than the right hemisphere.

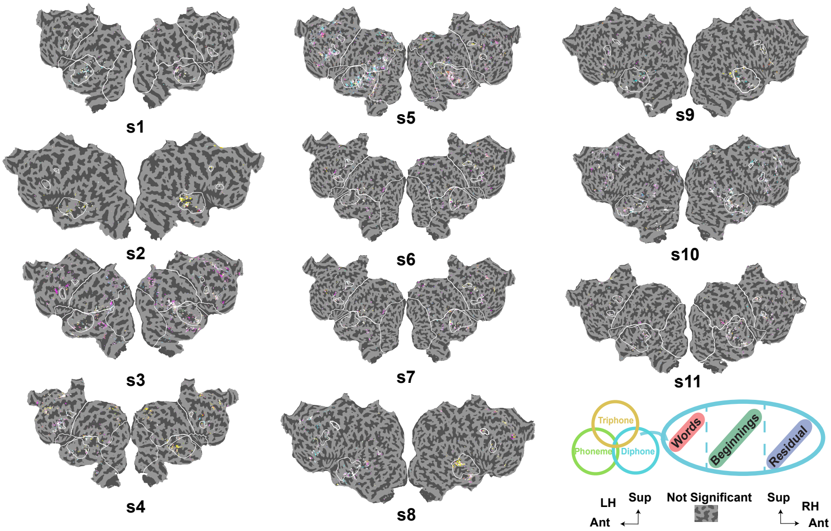

Fig. 8 Prediction performance of distinct diphone categories for 11 **subjects.** The cortical flatmaps show the significant prediction performance of the short words (red), word beginnings (green) and residuals (blue). The flatmaps did not show distinct cortical subregions within cortical areas with phonemic representations where the responses to short words were systematically higher than those to word beginnings of other diphones

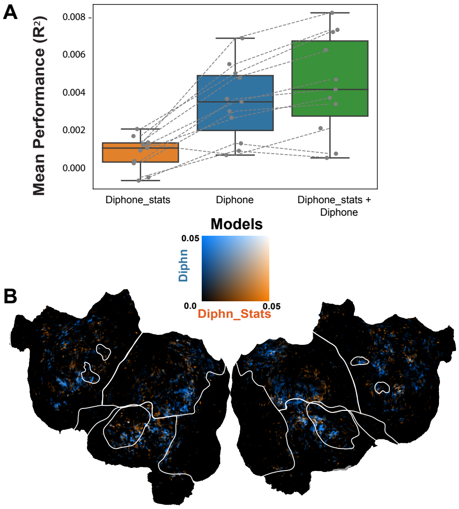

Supplementary Figure 9 Modeling on diphoneme statistical properties. In order to further explore if the cortical BOLD activities truly encode the content of diphones or merely the statistical properties of diphones, we built one diphone statistics model consisting of 8 phonological statistical features extracted from Irvine Phonotactic Online Dictionary [1]. These features describe the phonotactic probability of diphones (Supplementary Table 6). **Panel A** shows the average prediction performance of all the significant cerebral cortex voxels of 11 subjects (each dot represents one subject) from the diphone statistics model (orange), the diphone identity model (blue), and both models (green). It shows that the performance of the diphone statistics model is significantly lower than that of the diphone identity model (blue) ($t(1) = -30.19, p < 2.2^{10-16}$). All tests are two sided and corrected for multiple comparisons. The boxplots are defined the same way as in Figure 4 in the manuscript. **Panel B** shows the anatomical data for one example subject (S5). Blue voxels are better predicted by the diphone (content) model, and orange voxels are better predicted by the diphone statistics model. White voxels are equally well predicted by both models. This analysis shows that although diphone statistical properties can explain some of the variance of BOLD responses that could be captured by the diphone model, the actual diphone identities captured in the diphone model and not in the phonotactic probabilities yield significant additional explanatory power.

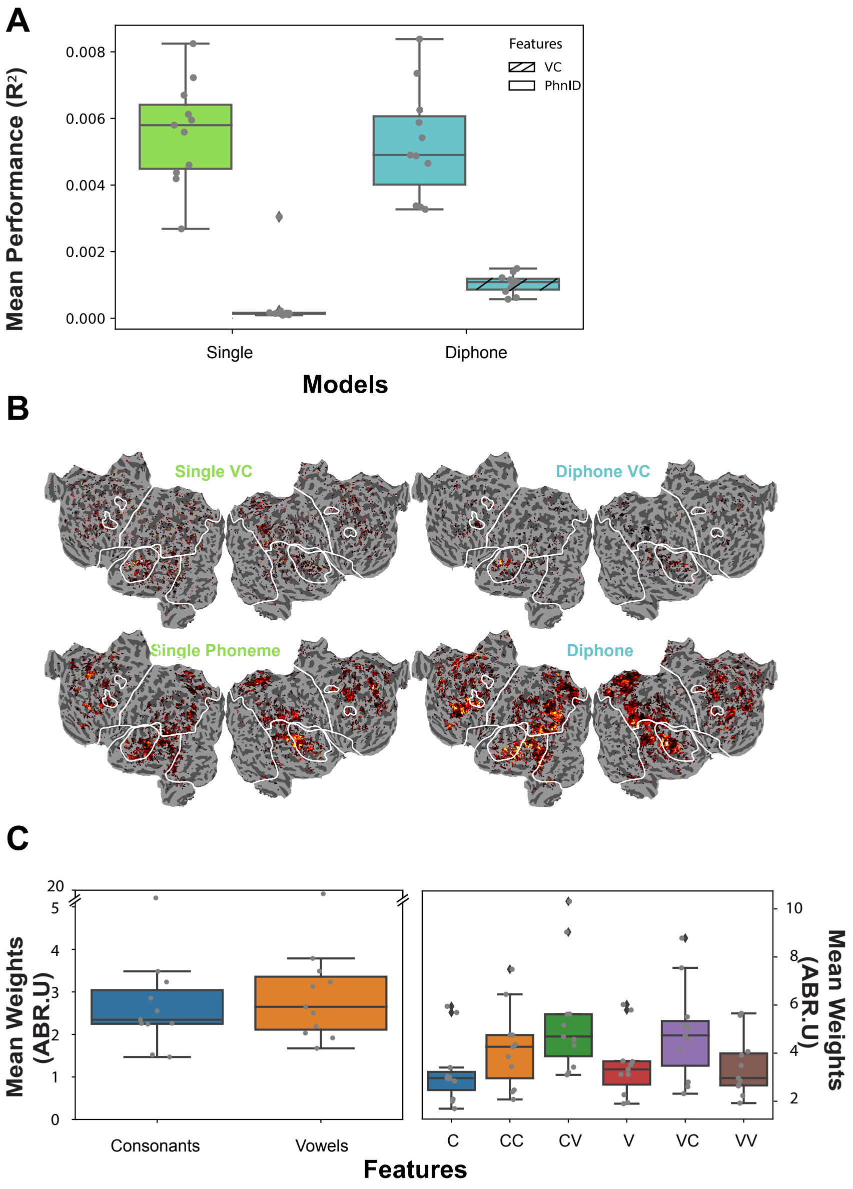

Supplementary Figure 10 Modeling on vowel and consonants. In order to explore how the properties of vowel and consonants contribute to the cerebral cortical encoding of phonemes and phonemic combinations, we calculated the prediction performance based on the identity of consonants and vowels of single phonemes and diphones (Supplementary Methods). **Panel A** shows that average prediction performance for each subject (each dot) for each model. **Panel B** shows the additive prediction performance of each model for each voxel on a flattened cortical map of subject 5. It illustrates that the phoneme identity models (single phoneme and diphone features) significantly outperform the vowel and consonants features. **Panel C** shows the average (across voxels and subjects) of the absolute value of the weights for each regressor/channel of the single vc and diphone vc features. There is no significant difference in the contribution consonants and vowels towards the single phoneme encoding. In addition, "vc" and "cv" combinations contribute the most to the diphone encoding. For both panel A and C, the statistics are derived from the performance of significant voxels (n $=2,409-11,668$ voxels) over 11 independent subjects. The boxplots are defined the same way as in Figure 4 in the manuscript.

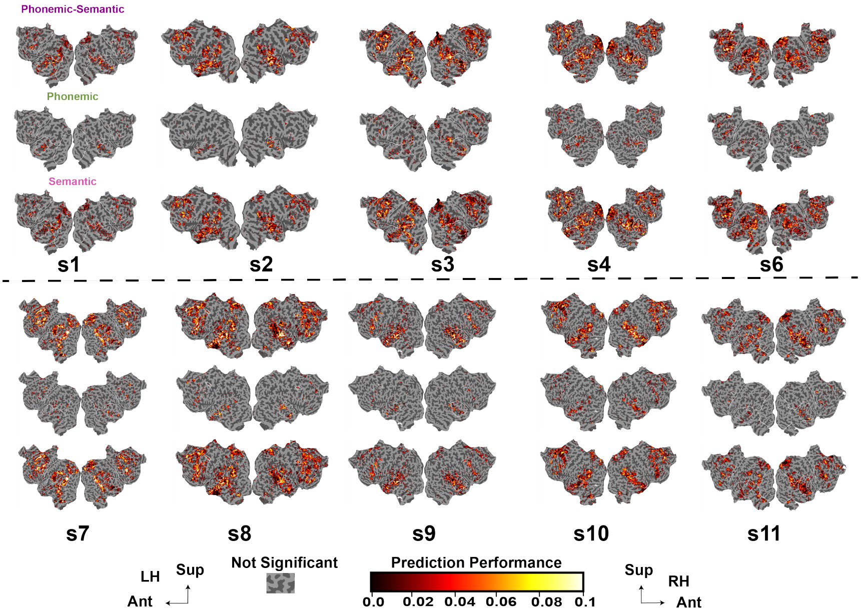

Fig. 11 Prediction performance of phonemic-semantic cortical maps for the remaining 10 subjects. The cortical flatmaps on the first row show the significant prediction performance of the Phonemic-Semantic VM model for all the subjects in the study (Subject 5 is shown on the main Figure 6). The Phonemic-Semantic VM uses the combination of the phonemic identities and semantic embeddings as features. The second and third row show the additive contribution of phonemic and semantic features to the Phonemic-Semantic VM.

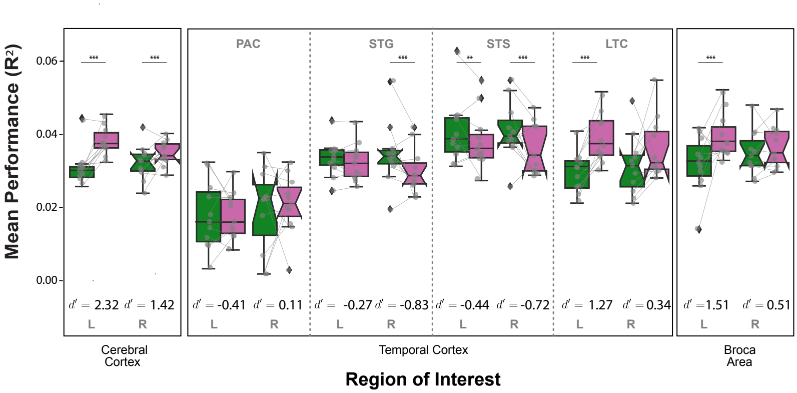

Supplementary Figure 12 Semantic vs phonemic contributions: hemispheric analysis. In order to examine whether the significant prediction performance from the phonemic model and semantic features are varied between left (box) and right (notched box) hemisphere across regions of interest, we performed a linear mixed-effect model with post-hoc Tukey test. The statistics are derived from the performance of significant phoneme-semantic voxels for each ROI ($n = 56 - 19,511$ voxels) over 11 independent subjects. All tests are two sided and corrected for multiple comparisons. The exact p values can be found in the Laterality of phonemic versus semantic representations section. The boxplots are defined the same way as in Figure 4 in the manuscript. In STG, phonemic model predicts significantly higher response in the right hemisphere, while it explains significantly more in STS of both hemispheres. In LTC and Broca's area, semantic features predict significantly higher response in the left hemisphere, while it explains significantly more variance of both cerebral cortical hemispheres. It indicates that the difference in semantic and phonemic processing is more prominent in the right hemisphere in STG and in the left hemiphere in LTC and Broca's area, while this difference is more balanced in the PAC and STS of both hemispheres.

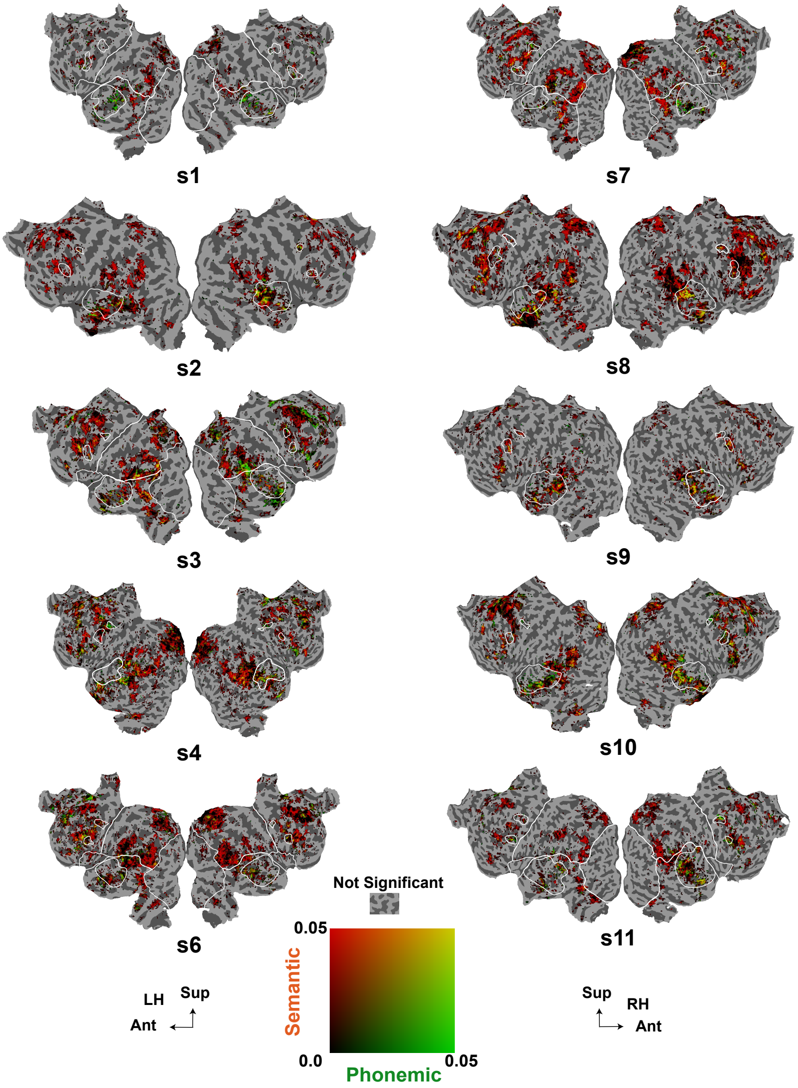

Supplementary Figure 13 Flatmap of individual subject for prediction performance of phonemic versus semantic based VM. The cortical flatmaps show the significant prediction performance of the phonemic VM versus semantic VM for all the subjects in the study (Subject 5 is shown on the main Figure 7). Green voxels are better predicted by phonemic model, while red voxels by semantic model. Yellow voxels share equally good prediction performance from phonemic and semantic models. These figures indicate two gradients of phonemic to semantic cortical representations. One in the temporal cortex, and another in the IPFC.

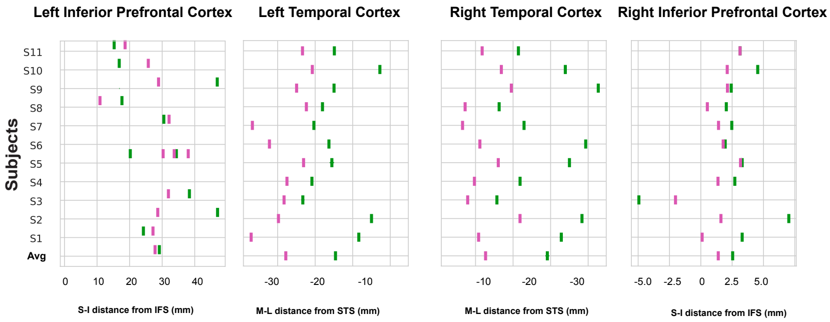

Supplementary Figure 14 Anatomical location of the center of mass for individual subjects. To quantify the phonemic to semantic transition, the center of mass of voxels in temporal cortex and IPFC in both hemispheres with significant prediction performance from phonemic versus semantic features for each subject is shown. It indicates that there is a medial/lateral gradient for phonemic to semantic transition in the temporal cortex of both hemispheres.

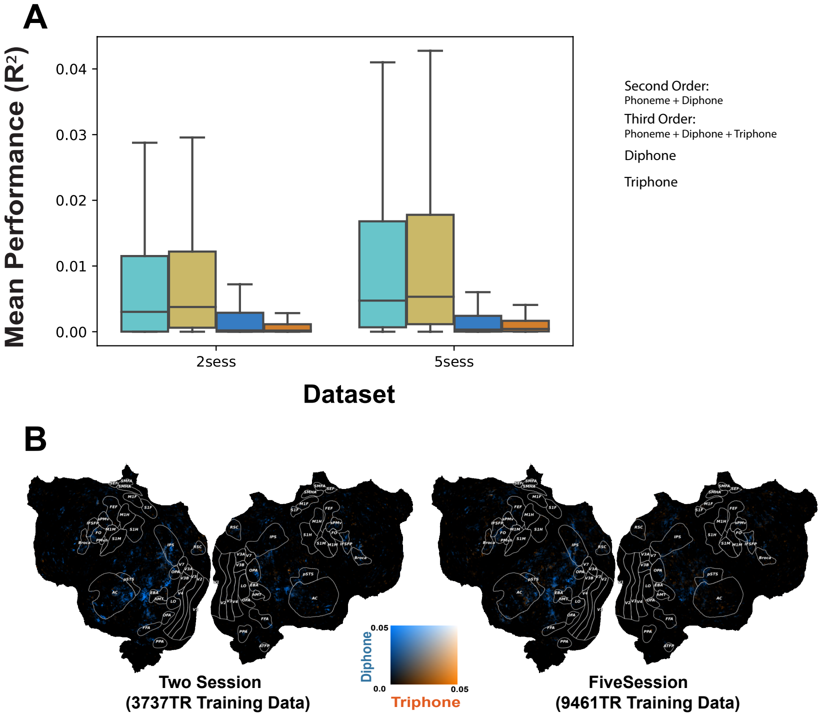

Supplementary Figure 15 Prediction Performance from five sessions of data. Panel A shows the average prediction performance (R^2) across significant voxels obtained from second order phonemic model (single phoneme + diphone: cyan), third order phonemic model (single phoneme + diphone + triphone: yellow), diphone only (blue) and triphone only (orange). The boxplots are defined the same way as in Figure 4 in the manuscript. It reveals that diphone features' prediction performance is significantly higher than the predictions obtained from triphone features (blue). This effect is observed both for analyses based on 2 sessions and 5 sessions of data collection. There is a small difference in terms of additional prediction obtained from the triphone features after taking into account diphone features when more data is used but our central result remains: the diphone features play a more important role than the triphone features to explain the bold response in phonemic cortical regions. **Panel B** shows the prediction performance of diphone features is dominant in both 2 sessions and 5 sessions data. The blue voxels are better explained by the diphone features, while the orange voxels are better explained by the triphone features. White voxels are equally well predicted by diphone and triphone features. The flatmap reveals more blue voxels than orange or white voxels irrespective of whether 2 or 5 sessions or data are used.

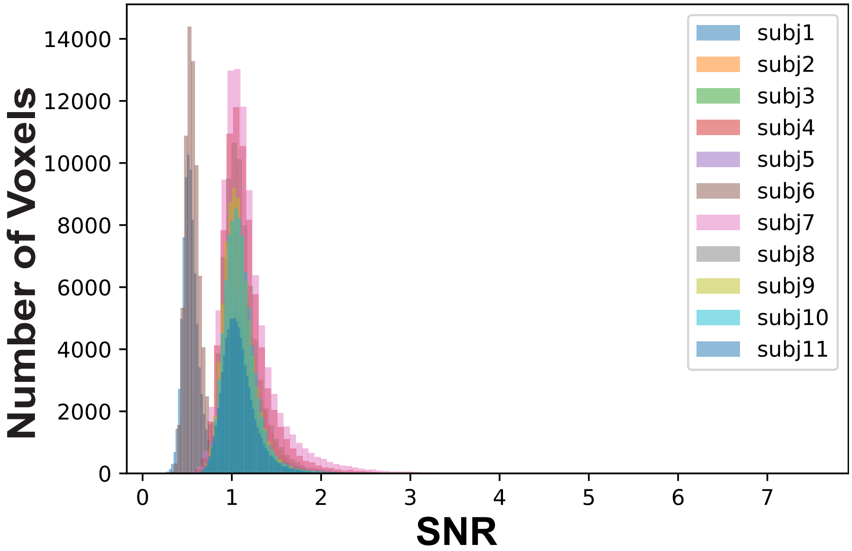

Supplementary Figure 16 Signal-to-noise ratio of BOLD signal for each subject. The SNR for each voxel of each subject is obtained from computing the coefficient of determination between two repeats of the BOLD responses collected when the subject was listening to the same story. The plot shows the histogram of SNR for each subject (each color represents one subject). It reveals that our simulation based on SNR=1 is representative to detect the phonemic feature sensitivity given our data size.

2 Supplementary Tables

Cerebral Cortex

subject	single	diphone	triphone	dprime
1	0.006	0.017	0.002	0.657
2	0.006	0.013	0.003	0.585
3	0.005	0.021	0.001	0.790
4	0.007	0.017	0.002	0.685
5	0.005	0.016	0.001	0.739
6	0.004	0.022	0.001	0.861
7	0.007	0.014	0.002	0.534
8	0.004	0.021	0.002	0.924
9	0.004	0.027	0.002	1.072
10	0.006	0.017	0.001	0.696
11	0.007	0.016	0.002	0.660
average	0.006	0.018	0.002	0.746
sem	0.000	0.001	0.000	0.047

Broca Area

subject	single	diphone	triphone	dprime
1	0.007	0.022	0.001	0.819
2	0.008	0.017	0.002	0.778
3	0.003	0.023	0.002	1.418
4	0.007	0.010	0.001	0.459
5	0.007	0.017	0.000	0.733
6	0.004	0.028	0.001	1.115
7	0.003	0.018	0.004	1.063
8	0.001	0.028	0.002	1.844
9	0.004	0.023	0.001	1.154
10	0.007	0.026	0.000	1.203
11	0.012	0.011	0.001	0.401
average	0.006	0.020	0.001	0.999
sem	0.001	0.002	0.000	0.127

Temporal Cortex

	PAC				STG				STS				LTC			
subject	single	diphone	triphone	dprime	single	diphone	triphone	dprime	single	diphone	triphone	dprime	single	diphone	triphone	dprime
1	0.015	0.000	0.000	-0.601	0.007	0.018	0.001	0.670	0.005	0.025	0.001	1.066	0.003	0.018	0.005	0.863
2	0.002	0.004	0.000	0.384	0.009	0.014	0.002	0.558	0.006	0.022	0.004	1.109	0.010	0.009	0.002	0.316
3	0.002	0.005	0.000	0.577	0.007	0.022	0.001	0.603	0.007	0.020	0.001	0.709	0.006	0.028	0.001	0.867
4	0.014	0.010	0.002	0.173	0.008	0.019	0.001	0.808	0.007	0.024	0.001	0.999	0.005	0.021	0.001	0.806
5	0.006	0.001	0.000	-0.252	0.004	0.020	0.001	0.824	0.006	0.017	0.001	0.750	0.005	0.010	0.002	0.512
6	0.003	0.015	0.002	0.848	0.006	0.018	0.000	0.611	0.003	0.031	0.001	1.233	0.003	0.021	0.002	0.938
7	0.017	0.005	0.001	-0.030	0.011	0.012	0.005	0.235	0.005	0.023	0.005	0.824	0.006	0.015	0.003	0.608
8	0.006	0.008	0.001	0.263	0.005	0.022	0.002	0.927	0.001	0.036	0.002	1.617	0.002	0.022	0.005	1.235
9	0.009	0.005	0.004	-0.137	0.005	0.029	0.002	0.962	0.003	0.040	0.002	1.512	0.004	0.024	0.002	1.217
10	0.009	0.010	0.001	0.287	0.007	0.022	0.001	0.800	0.006	0.027	0.001	1.042	0.005	0.024	0.001	1.219
11	0.009	0.008	0.004	0.141	0.011	0.013	0.002	0.477	0.010	0.020	0.001	0.757	0.005	0.011	0.002	0.635
average	0.008	0.006	0.001	0.150	0.007	0.019	0.002	0.680	0.005	0.026	0.002	1.056	0.005	0.018	0.002	0.838
sem	0.002	0.001	0.000	0.120	0.001	0.002	0.000	0.064	0.001	0.002	0.000	0.091	0.001	0.002	0.000	0.092

Supplementary Table 1 Table of prediction performance of phonemic processing in each ROI for each subject and statistics based on subject averages. The additive contribution of single phonemes, diphones and triphones to the prediction performance obtained with the Phonemic VM is shown for each subject for the entire cortex, Broca's area and the ROIs in the temporal cortex. The average and standard errors of estimated across subjects are shown in the last two rows. These data show that diphone prediction performance is significantly higher than single phoneme and triphones on average across cerebral cortex (11/11 subjects), Broca's area (9/11 subjects), STG (11/11 subjects), STS (11/11 subjects) and LTC (11/11 subjects). It indicates that the most important phoneme-related representations in the brain occur at the level of diphones. All tests are two sided. Yellow background: $p < 0.05$, Orange background: $p < 0.01$ and Red background: $p < 0.001$ obtained in a post-hoc pairwise t-test with Bonferroni correction.

Cerebral Cortex

subject	l_di_avg	l_sin_di	l_tri_di
1	1.589	0.614	2.565
2	1.399	0.294	2.504
3	2.220	1.229	3.211
4	1.574	0.745	2.403
5	1.901	0.715	3.087
6	2.145	1.029	3.260
7	1.430	0.463	2.396
8	2.041	1.128	2.953
9	2.405	1.781	3.029
10	1.684	0.465	2.903
11	1.665	0.774	2.557
average	1.823	0.840	2.806
sem	0.102	0.128	0.099

Broca's Area

subject	l_di_avg	l_sin_di	l_tri_di
1	1.367	0.431	2.303
2	1.445	1.099	1.792
3	2.649	2.996	2.303
4	0.714	-0.182	1.609
5	1.893	0.651	3.135
6	2.027	1.569	2.485
7	2.019	1.595	2.442
8	2.649	2.303	2.996
9	1.994	1.099	2.890
10	2.020	0.821	3.219
11	1.358	0.318	2.398
average	1.830	1.154	2.507
sem	0.174	0.277	0.157

Temporal Cortex

	PAC			STG			STS			LTC		
subject	l_single	l_diphone	l_triphone	l_single	l_diphone	l_triphone	l_single	l_diphone	l_triphone	l_single	l_diphone	l_triphone
1	-1.199	-2.398	-0.000	1.976	0.657	3.296	2.556	1.392	3.720	1.263	0.916	1.609
2	0.144	-0.405	0.693	1.472	0.359	2.584	2.447	1.407	3.486	0.795	-0.357	1.946
3	0.490	-0.405	1.386	1.979	0.609	3.350	2.013	0.721	3.305	2.497	1.433	3.561
4	0.450	-0.486	1.386	1.537	0.712	2.361	2.358	1.219	3.497	1.994	1.099	2.890
5	-0.602	-1.609	0.405	2.142	0.899	3.384	2.774	0.753	4.796	1.092	0.420	1.764
6	1.407	0.511	2.303	1.599	0.154	3.045	3.048	2.029	4.066	1.247	0.788	1.705
7	0.089	-1.075	1.253	0.670	-0.009	1.350	1.752	1.209	2.295	1.373	0.490	2.257
8	0.235	-0.916	1.386	1.786	1.237	2.335	3.314	2.621	4.007	1.966	1.764	2.169
9	0.405	-0.288	1.099	2.067	1.350	2.785	3.506	2.701	4.311	1.937	1.846	2.028
10	0.636	-0.336	1.609	1.899	0.819	2.979	2.400	1.216	3.584	1.893	1.435	2.351
11	0.182	-0.647	1.012	1.687	0.164	3.209	2.325	0.774	3.876	1.642	0.669	2.615
average	0.204	-0.732	1.139	1.710	0.632	2.789	2.590	1.459	3.722	1.609	0.955	2.263
sem	0.201	0.231	0.186	0.124	0.132	0.184	0.160	0.211	0.192	0.151	0.197	0.175

Supplementary Table 2 Table of voxel counts and corresponding statistics of phonemic processing in each ROI for each subject based on subject averages with logistic regression. In order to quantify the number of voxels that is best explained by single phoneme, diphone or triphone features, we assigned each cortical voxel to the best predictive feature. Then, we quantified the effect size of the difference in the average of the number of voxels best explained by each feature for each subject. This effect size is calculated as the average of the negative logits of the probability of being best explained by the single phone and triphone relative to the probability of being best diphone-based probability. The average and standard errors estimated across subjects are summarized as well. We performed a two-sided statistical analysis using mixed effect multinomial logistic regression which takes into account the varying number of voxels in each subject in its likelihood. This statistical analysis compares the actual probability of the number of voxels where the single phoneme, diphone and triphone contributions are higher to what is expected by chance given here by equal probability. We then use a likelihood ratio test comparing statistical mixed-effect model with fitted p_{single} and $p_{triphone}$ to the equal probability p_{single}=$p_{triphone}$=1/3. On average across the whole cerebral cortex, the additive prediction from the diphone features was consistently higher than that of the single phoneme or triphone features with $l = 1.802 + -0.172(2SE)$ ($\chi^2(2) = 1012.22, p < 2.2 \times 10^{-16}$, 11/11 subjects.Yellow background: $p < 0.05$, Orange: $p < 0.01$ and Red: $p < 0.001$ is obtained in a post-hoc pairwise t-test with Bonferroni correction). In the temporal cortex, the probabilities were estimated using a multinomial mixed effect statistical model with the subject as the random effect and the temporal cortex ROIs as a fixed effect (four levels: AC, STG, STS, and LTC). This statistical model was compared to the mixed effect multinomial model that did not include ROIs as fixed effect but did include the two non zero intercepts (yielding estimates for p_{single} and $p_{triphone}$ distinct from 1/3) and subject as a random effect. This interaction was significant ($\chi^2(6) = 227.88, p < 2.17 \times 10^{-46}$, 9/11 subjects) and showed a systematic increase in effect size from AC to STG and STS: the single phoneme, diphone and triphone-based models are equally good in AC ($l = 0.391 + -0.304(2SE)$, 4/11 subjects) but the diphone-based model becomes increasingly more and more dominant in STG ($l = 1.600 + -0.229(2SE)$, 11/11 subjects) and STS ($l = 2.423 + -0.223(2SE)$, 11/11 subjects) and less so in LTC ($l = 1.852 + -0.236(2SE)$, 9/11 subjects). This indicates that the additive contribution of the diphone feature is higher than the contribution of single phonemes and triphones in STG and STS but not in AC or LTC. In Broca's area, the additive prediction from the diphone feature was consistently higher than that of the single phoneme or triphone feature with $l = 1.790 + -0.305(2SE)$ ($\chi^2(2) = 287.76, p < 2.2 \times 10^{-16}$, 9/11 subjects). In sum, these results are in line with those obtained giving equal weighting to all subjects and with the results also obtained using the actual predicted value shown in Figure 4 of the main paper.

Diphone Category

subject	Words	Beginning	Residual
1	0.036	0.027	0.013
2	0.063	0.024	0.009
3	0.057	0.022	0.015
4	0.049	0.021	0.014
5	0.050	0.019	0.009
6	0.035	0.017	0.011
7	0.060	0.020	0.016
8	0.092	0.033	0.019
9	0.068	0.021	0.012
10	0.040	0.019	0.015
11	0.073	0.022	0.012
average	0.057	0.022	0.013
sem	0.005	0.001	0.001

Supplementary Table 3 Table of prediction performance of each diphone category for each subject. The contribution to the prediction of short words is significantly higher than the beginning of words and diphone residuals for the subjects with red background in the table. The average and standard errors of estimated across subjects are shown in the last two rows. Yellow background: $p < 0.05$, Orange background: $p < 0.01$ and Red background: $p < 0.001$ obtained in a post-hoc pairwise two sided t-test with Bonferroni correction.

Cerebral Cortex

subject	phonemic	semantic	dprime
1	0.030	0.030	-0.002
2	0.032	0.035	0.080
3	0.032	0.034	0.083
4	0.029	0.036	0.209
5	0.025	0.035	0.368
6	0.030	0.033	0.100
7	0.029	0.039	0.295
8	0.032	0.042	0.289
9	0.043	0.040	-0.065
10	0.031	0.034	0.122
11	0.029	0.036	0.281
average	0.031	0.036	0.160
sem	0.001	0.001	0.042

Broca Area

subject	phonemic	semantic	dprime
1	0.036	0.032	-0.142
2	0.038	0.042	0.196
3	0.034	0.042	0.311
4	0.022	0.038	0.628
5	0.029	0.032	0.139
6	0.035	0.031	-0.159
7	0.029	0.037	0.255
8	0.035	0.037	0.064
9	0.034	0.038	0.135
10	0.039	0.049	0.349
11	0.027	0.033	0.303
average	0.033	0.037	0.189
sem	0.002	0.002	0.068

Temporal Cortex

	PAC			STG			STS			LTC		
subject	phonemic	semantic	dprime	phonemic	semantic	dprime	phonemic	semantic	dprime	phonemic	semantic	dprime
1	0.015	0.005	-0.639	0.032	0.027	-0.176	0.039	0.027	-0.420	0.029	0.038	0.338
2	0.008	0.017	0.741	0.034	0.033	-0.056	0.049	0.042	-0.243	0.028	0.032	0.181
3	0.009	0.012	0.223	0.036	0.025	-0.317	0.035	0.032	-0.094	0.041	0.041	-0.009
4	0.032	0.020	-0.600	0.035	0.030	-0.156	0.037	0.040	0.101	0.032	0.040	0.229
5	0.007	0.014	0.713	0.026	0.025	-0.046	0.027	0.032	0.229	0.020	0.034	0.562
6	0.022	0.028	0.235	0.026	0.025	-0.058	0.038	0.033	-0.155	0.029	0.036	0.226
7	0.025	0.019	-0.228	0.034	0.029	-0.135	0.042	0.030	-0.359	0.031	0.036	0.163
8	0.017	0.017	-0.006	0.032	0.026	-0.235	0.043	0.030	-0.497	0.032	0.053	0.530
9	0.022	0.022	-0.001	0.049	0.042	-0.194	0.058	0.050	-0.191	0.038	0.037	-0.025
10	0.023	0.029	0.182	0.035	0.038	0.110	0.040	0.046	0.177	0.038	0.032	-0.225
11	0.024	0.025	0.027	0.032	0.032	0.002	0.038	0.039	0.026	0.022	0.032	0.469
average	0.018	0.019	0.059	0.034	0.030	-0.115	0.041	0.036	-0.130	0.031	0.037	0.222
sem	0.002	0.002	0.134	0.002	0.002	0.036	0.002	0.002	0.073	0.002	0.002	0.074

Supplementary Table 4 Table of prediction performance performance of Phonemic-Semantic processing in each ROI for each subject and statistics based on subject averages. The table shows the mean prediction performance of each subject's BOLD response explained by the third order phonemic and semantic based VM for each ROI (whole cortex, AC, STG, STS, LTC, and Broca area). The average and standard errors of the mean performance estimated across subjects for each ROI are presented in the last two rows. These data show that semantic prediction performance is significantly higher than phonemic features on average across cerebral cortex (9/11 subjects), Broca's area (4/11 subjects), and LTC (5/11 subjects), while phonemic prediction performance is higher than the semantic features in STG (3/11 subjects) and STS (6/11 subjects). Yellow background: $p < 0.05$, Orange background: $p < 0.01$ and Red background: $p < 0.001$ obtained in a post-hoc pairwise two sided t-test with Bonferroni correction.

Count Data for Phonemic VS Semantic Comparisons

subject	l_phnSem_APC	l_phnSem_STG	l_phnSem_STS	l_phnSem_LTC	l_phnSem_BA	l_phnSem_CC
1	-0.588	-0.729	-1.200	0.833	-0.105	0.288
2	-0.182	-1.109	-0.736	0.226	0.693	0.270
3	-0.693	-0.726	-0.366	0.829	1.504	0.634
4	-0.480	-0.444	0.048	0.864	-0.542	0.742
5	0.596	-0.217	-0.557	0.654	0.087	1.049
6	0.642	-0.329	-1.128	1.995	0.288	1.213
7	0.359	0.134	0.654	1.363	0.606	1.336
8	0.074	0.231	0.366	0.338	0.211	0.459
9	0.288	0.660	0.928	1.295	2.451	1.514
10	0.457	0.171	0.134	1.596	-0.128	0.864
11	0.095	0.167	0.382	0.758	0.591	0.437
average	0.052	-0.199	-0.134	0.978	0.514	0.801
sem	0.143	0.159	0.215	0.161	0.253	0.130

Supplementary Table 5 Table of voxel counts and corresponding statistics of Phonemic-Semantic processing in each ROI for each subject based on subject averages with logistic regression. In order to quantify the number of voxels that is best explained by phonemic or semantic model, we assigned each cortical voxel to the best predictive feature. Then, we quantified the effect size of the difference by calculating the negative logit of the probability based on the number of voxels being best explained by the Phonemic features vs the additive contribution of the semantic feature in those significant voxels (winner-take-all count analysis). The average and standard errors of the logits estimated across subjects for each ROI are presented in the last two rows. Using subject mean and two SE, one could conclude that Semantic VM is more predictive in the whole cortex, in LTC and in Broca's area (just reaching the threshold of significance). To better model the statistical significance for this count data, we also used mixed-effect logistic regression, which incorporates the number of voxels in each subject in its likelihood. In logistic regression, we statistically assessed whether the number of voxels best explained by the phonemic vs semantic features are different from expectations based on the binomial distribution. The number of voxels best explained by semantic versus phonemic features was found to be significantly higher throughout the whole cortex with the logit equal to $l = 0.159 + -0.106(2SE)$ (Likelihood ratio test comparing statistical mixed-effect model with equal probability of semantic and third order phonemic voxels: $\chi^2(1) = 6.61, p = 1.0 \times 10^{-2}$). The number of voxels best explained by the semantic based VM was also higher in Broca's area: $l = 0.598 + -0.266(2SE)$ ($\chi^2(1) = 11.51, p = 6.9 \times 10^{-4}$). In order to further examine how different temporal cortical areas involved in the phonemic versus semantic processing, we used a generalized mixed-effect linear statistical model (glmer with family=binomial) with the temporals cortex ROIs (four levels: PAC, STG, STS, and LTC) as regressor, subject as the random effect and the fraction of voxels best explained by the semantic feature as the response variable. We found that the fraction of voxels best explained by the semantic based VM varied significantly across ROIs (likelihood ratio test with nested model that does not include ROIs, $\chi^2(3) = 583.80, p < 2.2 \times 10^{-16}$). Moreover, the number of voxels best predicted by the semantic feature is significantly higher in LTC $l = 0.244 + -0.068(2SE)$ ($\chi^2(1) = 19.21, p = 1.2 \times 10^{-5}$), but the differences are negligible in STG $l = -0.070 + -0.160(2SE)$ ($\chi^2(1) = 0.74, p = 3.9 \times 10^{-1}$) and STS $l = 0.293 + -0.240(2SE)$ ($\chi^2(1) = 4.78, p = 2.9 \times 10^{-2}$). These results are in line with those obtained giving equal weighting to all subjects and with the results also obtained using the actual predicted value shown in Figure 6.

Feature Name	Feature Definition
unsDPAV	Unstressed diphone probability average; vowel-stress ignored
unsFDPAV	unsDPAV weighted with SUBTLEXus word frequency
unsLDPAV	unsDPAV weighted with log SUBTLEXus word frequency
unsCDPAV	unsDPAV weighted with SUBTLEXus context count
strDPAV	Stressed diphone probability average; distinct stressed-vowels
strFDPAV	strDPAV weighted with SUBTLEXus word frequency
strLDPAV	strDPAV weighted with log SUBTLEXus word frequency
strCDPAV	strDPAV weighted with SUBTLEXus context count

Supplementary Table 6 Table of definition of diphone phontactic probability features. The table summarizes the name and content of each feature for modeling diphone phontactic probability. Phonotactic probabilities refer to the concurrence likelihood of the sequence of sounds that are present in a given word [1, 2]. Diphone probability average refers to the average likelihood of each diphone occurring in each position of a word. In these measures, the syllable stress placement of vowels can also be considered. For stressed calculations, identical vowel sounds are considered to be distinct phonemes depending on primary, secondary, or no-stress placement. In unstressed calculations, vowel sounds are considered to be single phoneme categories.

3 Supplementary Methods

3.1 Feature Construction

In order to explore how the properties of vowel and consonants contribute to the cerebral cortical encoding of phonemes and phonemic combinations, we created two features based on the identity of consonants and vowels of each phoneme.

Single-vc features uses a one-hot coding matrix (dimension: [number of TR, 2]) to encode if a single phoneme is a consonant or vowel. Diphone-vc features (dimension: [number of TR, 6]) encodes if a diphone belongs to any of these six consonant/vowel combinations: vowel-vowel (vv), vowel-consonant (vc), consonant-vowel (cv) and consonant-consonant (cc), vowel-blank (vb), consonant-blank (cb)

Afterwards, We then fitted four nested VMs: single vc model (using single-vc feature), first order vc model (using single-vc + single phoneme features), first order vc + diphone vc model (using single-vc + single phoneme + diphone-vc features) and second order vc model (using single-vc + single phoneme + diphone-vc + diphone features). The variance explained by the single-vc feature is obtained from the single vc model, while the additional variance explained by diphone-vc feature is obtained from subtracting the explainable variance of the first order vc model from the explainable variance of the first order vc + diphone vc model. In addition, the additional variance explained by the single phoneme identities beyond vowel and consonant categories is obtained from subtracting the explainable variance of the single vc model (using single-vc feature) from the first order vc model. Similarly, the variance explained by the diphone identities beyond the combination of vowel and consonants is obtained from subtracting the explainable variance of the first order vc + diphone vc model from the second order vc model.

Furthermore, In order to compare the relative contribution of vowels and consonants towards the phoneme encoding, we examined the modeling weights (coefficients) of single-vc (consonant or vowels) and diphone-vc (vv, vc, cv, cc, vb, cb) features. The weights of each voxel have been scaled by the prediction performance of this voxel in order to get rid of the random effects from noisy voxels.

4 Supplementary Notes

4.1 Laterality of the phonemic representation and segmentation.

We examined whether the phonemic representation was lateralized. First, we found that the average performance of the full *phonemic* was significantly different throughout the cerebral cortex across the right and left hemispheres (likelihood ratio test with the nested statistical model that does not include the hemisphere factor: $\chi^2(1) = 5.07, p = 2.4 \times 10^{-2}$), as well as in the LTC ($\chi^2(1) = 13.52, p = 2.4\times10^{-4}$). The prediction performance was not lateralized in AC ($\chi^2(1) = 0.27, p = 6.0 \times 10^{-1}$), STG ($\chi^2(1) = 0.26, p = 6.1 \times 10^{-1}$), STS($\chi^2(1) = 0.04, p = 8.5 \times 10^{-1}$) or Broca's area ($\chi^2(1) = 0.05, p = 8.2 \times 10^{-1}$).

We also tested whether the segmentation at the level of the diphone was comparable for the left and right hemispheres (Supplementary Fig 7). The unique contributions of the three levels of phonemic segmentation were significantly different between left and right hemispheres when the entire cortex is analyzed ($\chi^2(2) = 12.32, p = 2.1 \times 10^{-3}$), as well as for the temporal cortex ($\chi^2(12) = 78.74, p = 7.2\times10^{-12}$) and for Broca's area ($\chi^2(2) = 44.55, p = 2.1\times 10^{-10}$). More specifically, within the temporal cortex, the additive contributions based on three levels of phonemic segmentation do not differ significantly between left and right STS ($\chi^2(2) = 4.23, p = 0.12$), but do differ significantly in left and right primary auditory cortex ($\chi^2(2) = 36.63, p = 1.1 \times 10^{-8}$), STG ($\chi^2(2) = 28.25, p = 7.3 \times 10^{-7}$), and LTC ($\chi^2(2) = 14.57, p = 6.9 \times 10^{-4}$). In STG, STS, LTC and Broca's area, the effect size d' was higher in the left versus right hemispheres. This indicates that the diphone segmentation for phonemic processing is more prominent in the left hemisphere than the right hemisphere.

4.2 Laterality of phonemic versus semantic representations.

We also examined whether the semantic representation was lateralized. First, we found that the average performance of the the semantic VM was significantly different throughout the cerebral cortex across the right and left hemispheres ($\chi^2(1) = 255.53, p < 2.2 \times 10^{-16}$), as well as in the STG ($\chi^2(1) = 9.57, p = 2.0 \times 10^{-3}$), STS ($\chi^2(1) = 5.76, p = 1.6 \times 10^{-2}$), and Broca's area ($\chi^2(1) = 5.09, p = 2.4 \times 10^{-2}$). The prediction performance was not lateralized in LTC ($\chi^2(1) = 0.86, p == 3.5 \times 10^{-1}$)

Next, we examined whether the relative predictive power of the phonemic versus semantic VM varied significantly across hemispheres (Supplementary Fig 12). We found that this interaction was statistically significant for the whole cortex (mixed-effect statistical models with subject as random factor: likelihood ratio test with the nested statistical model that does not include the interaction between hemisphere and models: $\chi^2(1) = 93.12, p < 2.2 \times 10^{-16}$) and in the temporal cortex (likelihood ratio test with the nested statistical

model that does not include ROIs, features spaces, hemisphere and the interaction between ROIs and feature spaces: $\chi^2(7) = 32.32, p = 3.5 \times 10^{-5}$), but not for Broca's area ($\chi^2(1) = 1.76, p = 1.8 \times 10^{-1}$). In addition, within the temporal cortex, hemispheric differences in relative performance were not significant in primary AC ($\chi^2(1) = 0.98, p = 3.2 \times 10^{-1}$) and STS ($\chi^2(1) = 1.36, p = 2.4 \times 10^{-1}$), but significant in STG ($\chi^2(1) = 5.06, p = 2.5 \times 10^{-2}$), and LTC ($\chi^2(1) = 10.42, p = 1.2 \times 10^{-3}$). This indicates that the difference in semantic and phonemic processing is more prominent in the right hemisphere in STG and LTC, while this difference is more balanced in the PAC, STS and Broca's area of both hemispheres. Supplemental figure 12 shows the data for all subjects and the effect sizes across two hemispheres between the semantic and phonemic based VMs.

Supplementary References

[1] Vaden KI, Halpin H, Hickok GS (2009) Irvine phonotactic online dictionary, version 2.0.[data file]

[2] Vitevitch MS, Luce PA (2004) A web-based interface to calculate phonotactic probability for words and nonwords in english. Behavior Research Methods, Instruments, & Computers 36(3):481–487
