# Supplementary material for: Phonemic segmentation of narrative speech in human cerebral cortex
Source: Nat Commun. 2023 Jul 18;14:4309. doi: 10.1038/s41467-023-39872-w (PMC10354060; doi:10.1038/s41467-023-39872-w)
Supplement: Supplementary file 3 — Reporting Summary [file 41467_2023_39872_MOESM3_ESM.pdf]

nature portfolio

Corresponding author(s): Frédéric Theunissen

Last updated by author(s): May 9, 2023

Reporting Summary

Nature Portfolio wishes to improve the reproducibility of the work that we publish. This form provides structure for consistency and transparency in reporting. For further information on Nature Portfolio policies, see our Editorial Policies and the Editorial Policy Checklist.

Statistics

For all statistical analyses, confirm that the following items are present in the figure legend, table legend, main text, or Methods section.

n/a	Confirmed	
☐	☒	The exact sample size (*n*) for each experimental group/condition, given as a discrete number and unit of measurement
☐	☒	A statement on whether measurements were taken from distinct samples or whether the same sample was measured repeatedly
☐	☒	The statistical test(s) used AND whether they are one- or two-sided<br>*Only common tests should be described solely by name; describe more complex techniques in the Methods section.*
☐	☒	A description of all covariates tested
☐	☒	A description of any assumptions or corrections, such as tests of normality and adjustment for multiple comparisons
☐	☒	A full description of the statistical parameters including central tendency (e.g. means) or other basic estimates (e.g. regression coefficient) AND variation (e.g. standard deviation) or associated estimates of uncertainty (e.g. confidence intervals)
☐	☒	For null hypothesis testing, the test statistic (e.g. *F*, *t*, *r*) with confidence intervals, effect sizes, degrees of freedom and *P* value noted<br>*Give P values as exact values whenever suitable.*
☒	☐	For Bayesian analysis, information on the choice of priors and Markov chain Monte Carlo settings
☒	☐	For hierarchical and complex designs, identification of the appropriate level for tests and full reporting of outcomes
☐	☒	Estimates of effect sizes (e.g. Cohen's *d*, Pearson's *r*), indicating how they were calculated

Our web collection on statistics for biologists contains articles on many of the points above.

Software and code

Policy information about availability of computer code

Data collection	MRI data was collected using syngoMR software distributed by Siemens (versions 15 and 17A). The stimuli in these experiments were pre-recorded stories from the Public Radio Exchange (PRX) radio show “The Moth Radio Hour”, which were presented with python 2.7.
Data analysis	Python 3.6 was used to preprocess the MRI data, making use of FSL(5.3: FLIRT and MCFLIRT) and Freesurfer (5.3). Python 3.6 was used to analyze the data, which primarily relied on pycortex (version 1.3.0; https://github.com/gallantlab/pycortex/), cottoncandy (version 0.2.0; https:// github.com/gallantlab/cottoncandy), and himalaya (version 0.4.2; https://github.com/gallantlab/himalaya).<br><br>Custom code used for analyses is available at github repo (https://github.com/theunissenlab/phoneme_segmentation) with DOI: 10.5281/zenodo:7938599

For manuscripts utilizing custom algorithms or software that are central to the research but not yet described in published literature, software must be made available to editors and reviewers. We strongly encourage code deposition in a community repository (e.g. GitHub). See the Nature Portfolio guidelines for submitting code & software for further information.

Data

Policy information about availability of data

All manuscripts must include a data availability statement. This statement should provide the following information, where applicable:

- Accession codes, unique identifiers, or web links for publicly available datasets
- A description of any restrictions on data availability
- For clinical datasets or third party data, please ensure that the statement adheres to our policy

Data has been made available online (https://gin.g-node.org/gallantlab/story_listening) with DOI:10.12751/g-node.wf9cbv. All data other than anatomical brain images has been be shared (as there is concern that anatomical images could violate subject privacy). However, we have also provided matrices that map from volumetric data to cortical flatmaps for visualization purposes.

Research involving human participants, their data, or biological material

Policy information about studies with human participants or human data. See also policy information about sex, gender (identity/presentation), and sexual orientation and race, ethnicity and racism.

Reporting on sex and gender	Seven male subjects (S1: age 26 , S2: age 31 , S5: age 30, S6: age 25, S7: age 36, S9: age 24, S10: age 24)<br>Four female subjects (S3: age 28, S4: age 25, S8: age 24, S11: 31).
Reporting on race, ethnicity, or other socially relevant groupings	The race and ethnicity of the subjects was representative of the diverse student population of the UC Berkeley campus (white, Asian, Latino). The social-economic status of the students was relatively homogeneously high since they all had some post-graduate education. Since our study was not based on dividing subjects into separate groups, there were no confounding variables and the level of social groups. Mixed-effect modeling was used to correct for potential confounding effects at the level of the single subject.
Population characteristics	The participants were all young healthy adults aged 24-36 who belonged to the UC Berkeley research community (graduate students, post-docs).
Recruitment	The subjects were recruited via announcements to the neuroscience community at UC Berkeley. The subjects participated in the study as volunteers. Subjects had to be native speakers of American English and have normal hearing.
Ethics oversight	The use of human subjects in this study was approved by the UC Berkeley Committee for the Protection of Human Subjects. A written statement of informed consent has been obtained from each subject.

Note that full information on the approval of the study protocol must also be provided in the manuscript.

Field-specific reporting

Please select the one below that is the best fit for your research. If you are not sure, read the appropriate sections before making your selection.

☒ Life sciences ☐ Behavioural & social sciences ☐ Ecological, evolutionary & environmental sciences

For a reference copy of the document with all sections, see nature.com/documents/nr-reporting-summary-flat.pdf

Life sciences study design

All studies must disclose on these points even when the disclosure is negative.

Sample size	We did not perform an explicit power analysis for number of subjects. Instead, the number of subjects was chosen based on results obtained in prior studies. In a prior study, data obtained from 5 subjects were sufficient to distinguish brain regions that represented articulatory features of speech above and beyond what would be expected from sensitivity to auditory features and semantic features. Using similar criteria for generating cortical functional maps and assuming similar effect sizes but for an application that required finer spatial resolution (approximately dividing areas in half), we reasoned that a sample size of approximately 10 subjects would be sufficient. Our statistical analyses (either at the subject level or by accounting for subject differences in mixed-effect modeling) showed that, indeed, we were able to delineate the finer cortical maps involved in the phonemic segmentation with our 11 subjects.<br><br>Note that the duration of the BOLD recordings for each subject is just as critical as number subjects (if not more) to assess the power of our analyses. For this purpose we performed a series of simulations (included in the supplemental material) that can be directly compared to power analyses in more classical analyses. In these simulations, we test specific effect sizes (in the size of the coefficients of the linear filters yielding a predictive signal for the BOLD recordings obtained in single voxels) and assess whether it can be detected given a particular duration for the recording time. We believe that this "power analysis" can serve as an example for other studies that fit linear dynamical models to describe the stimulus-response function explaining the BOLD signal.
Data exclusions	No data were excluded.
Replication	Replication was enforced at the subject and single voxel level by using two-level cross-validation. First, using 10-fold cross-validation, we fitted the parameters of voxel-wise linear models we trained the model in a subset of data (10 stories). Second , actual model performance

	was assessed by testing the model on an 11th story reserved solely for this purpose. Permutation tests were then used to assess the significance of predictions beyond expected baseline.<br>Replication was also verified across subjects, by performing all the analyses at the level of a single subject but reporting (by quantification and statistical modeling) only the effects observed across all (or a large majority) of subjects.
Randomization	In this study, all subjects belong to the same group (healthy adults) and are presented with the same stimulus set. The order of the stimulus presentation is randomized.
Blinding	There are no distinct groups in this study. The data from all subjects is analyzed using the same software, algorithms and settings.

Reporting for specific materials, systems and methods

We require information from authors about some types of materials, experimental systems and methods used in many studies. Here, indicate whether each material, system or method listed is relevant to your study. If you are not sure if a list item applies to your research, read the appropriate section before selecting a response.

Materials & experimental systems

n/a	Involved in the study
☒	☐ Antibodies
☒	☐ Eukaryotic cell lines
☒	☐ Palaeontology and archaeology
☒	☐ Animals and other organisms
☒	☐ Clinical data
☒	☐ Dual use research of concern
☒	☐ Plants

Methods

n/a	Involved in the study
☒	☐ ChIP-seq
☒	☐ Flow cytometry
☐	☒ MRI-based neuroimaging

Magnetic resonance imaging

Experimental design

Design type	Naturalistic stimuli listening experiment.
Design specifications	The stimuli in these experiments were pre-recorded stories from the Public Radio Exchange (PRX) radio show “The Moth Radio Hour”, which has been used in previous studies of our lab (Huth et al, 2016; de Heer et al, 2017; Deniz et al, 2019). These sounds have been annotated for their word and phonetic content. They are engaging stories that capture the attention of the subjects. Each scanning sessions lasted approximately 2.5 hours and included time needed to play the audio/visual stimuli used as generic localizers and two three-hour sessions during which the subjects listened to Moth stories.The stimuli were split into separate model estimation and model validation sets. The model estimation stimulus-set consisted of ten 10– to 15–minute stories played once each. The length of each scan was tailored to the story and also included 10 seconds of silence both before and after the story. Each subject heard the same 10 stories, 5 of which were told by male speakers and 5 by female speakers. The model validation stimulus-set consisted of a single 10–minute story told by a female speaker that was played twice for each subject in order to estimate voxel response reliability and noise ceiling.
Behavioral performance measures	Each subject’s handedness was evaluated by the Edinburgh handedness inventory.

Acquisition

Imaging type(s)	functional MRI
Field strength	3T
Sequence & imaging parameters	Structural MRI data and blood oxygen level dependent (BOLD) fMRI responses from each subject were obtained while they listened to approximately 2 hours and 20 minutes of natural stories. For nine of the subjects, these data were collected during two separate scanning sessions that lasted no more than 2 hours each. For two of the subjects (S1 and S5) the validation data (two repetitions of a single story) were collected in a third, separate session. MRI data were collected on a 3T Siemens TIM Trio scanner at the UC Berkeley Brain Imaging Center, using a 32-channel Siemens volume coil. Functional scans were collected using a gradient echo-EPI sequence with repetition time (TR) = 2.0045s, echo time (TE) = 31ms, flip angle = 70degrees, voxel size = 2.24x2.24x4.1 mm, matrix size = 100x100, and field of view = 224x224 mm. 32 axial slices were prescribed to cover the entire cortex. A custom-modified bipolar water excitation radiofrequency (RF) pulse was used to avoid signals from fat tissue. Anatomical data were collected using a T1-weighted MP-RAGE sequence on the same 3T scanner.
Area of acquisition	Whole cerebral cortex was imaged.

Diffusion MRI ☐ Used ☒ Not used

Preprocessing

Preprocessing software	Customized codes based on FSL5.3
Normalization	Each functional run was motion-corrected using the FMRIB Linear Image Registration Tool (FLIRT) from FSL 4.2 (Jenkinson and Smith, 2001). All volumes in the run were then averaged to obtain a high quality template volume.
Normalization template	FLIRT was also used to automatically align the template volume for each run to the overall template, which was chosen to be the template for the first functional run for each subject. These automatic alignments were manually checked and adjusted for accuracy. The cross-run transformation matrix was then concatenated to the motion-correction transformation matrices obtained using MCFLIRT, and the concatenated transformation was used to resample the original data directly into the overall template space.
Noise and artifact removal	Low-frequency voxel response drift was identified using a 2nd order Savitsky-Golay filter with a 120–second window, and this was subtracted from the signal. After removing this time-varying mean, the response was scaled to have unit variance (i.e. z-scored).
Volume censoring	No volume censoring was performed. Subjects were wearing customized headcases to prevent excessive movement.

Statistical modeling & inference

Model type and settings	Voxelwise Encoding Modeling, Variance Partition
Effect(s) tested	Linear mixed-effects statistical models with subjects as random effect were used to compare the model performance for different models, ROIs, and hemispheres. Cohen's dprime was used to quantify the effect size of the differences between different models for various ROIs and hemispheres.

Specify type of analysis: ☐ Whole brain ☐ ROI-based ☒ Both

Anatomical location(s)

Regions of interests including auditory cortex broadly defined (AC), Broca’s area, and ventral speech premotor area (sPMv) were defined based on standard functional localizer scans. Voxels that were responsive when the subject listened to 10 repetitions of a one-min auditory stimulus with 20 – sec seg- ments of music, speech and natural sound are considered to belong to AC. AC thus includes both primary and secondary auditory cortical areas. When the subject continuously subvocalizes self-generated sentences, the active voxels located at the triangular part of the inferior frontal gyrus are determined as Broca’s area and voxels located at the premotor cortex as sPMv. The repeata- bility of the voxels’ response was calculated as an F statistic given by the ratio of the total variance responses over the residual variance.

Other ROIs were defined based on speech processing relevant anatomical landmarks (Binder et al, 2009; Bookheimer, 2002; Hickok and Poeppel, 2007; Price, 2010; Huth et al, 2016) obtained from the structural MRI scan. In par- ticular, the inferior prefrontal cortex (IPFC) contains cortical regions ventral to inferior frontal sulcus (IFS), while superior prefrontal cortex (SPFC) con- tains regions dorsal to superior frontal sulcus (SFS). The peak of the superior parietal gyrus separates the lateral parietal cortex (LPC) from medial parietal cortex (MPC). LPC contains supramarginal gyrus (SMG) and angular gyrus (AG). The peak of the inferior temporal gyrus is used to separate the lateral temporal cortex (LTC) from ventral temporal cortex (VTC).

Statistic type for inference<br>(See Eklund et al. 2016)	For the significance of the coefficients of the voxel-wise modeling: cross-validation and permutation test. For mixed-effect tests for effects in ROI/hemispheres taking into account subject differences: likelihood ratio tests. For results first estimated for each subject and then compared across subjects using t-test with Bonferroni corrections or Fisher Exact tests for proportions.
Correction	The statistical significance of the cross-validated coefficient of determination R-square was estimated using a permutation analysis. First, We obtained a set of regularization hyperparameters from fitting each model for each subject. Then, a permuted R2 for each model of each subject was obtained by refitting the model using this set of hyperparameters and the shuffled regressors of each feature space within the model. This process was repeated 1, 000 times to generate the null distribution of cross-validated R2 for each voxel of each subject. Based on the values of this null distribution, we used variable thresholds yielding a significance level of 1% corrected using the False Discovery Rate (FDR) procedure to determine statistical significance.

Models & analysis

n/a	Involved in the study
☒	☐ Functional and/or effective connectivity
☒	☐ Graph analysis
☐	☒ Multivariate modeling or predictive analysis

Multivariate modeling and predictive analysis

Independent Variable/Stimuli & Train and test separation
The stimuli were split into separate model estimation and model validation sets. The model estimation stimulus-set consisted of ten 10– to 15–minute stories played once each. The length of each scan was tailored to the story and also included 10 seconds of silence both before and after the story. Each subject heard the same 10 stories, 5 of which were told by male speakers and 5 by female speakers. The model

validation stimulus-set consisted of a single 10–minute story told by a female speaker that was played twice for each subject in order to estimate voxel response reliability and noise ceiling. This resulted in 3737 time points (sampled at TR) for the training dataset and 291 time points for the validation dataset.

Feature space and model construction

In order to localize the phonemic brain regions, to investigate phonemic segmentation and the phoneme to word/meaning transitions, we constructed six distinct feature spaces: time-varying power spectrum, phoneme rate, single phoneme, diphone, triphone, and semantic features.

Voxelwise model fitting and validation

Based on these feature spaces, we created different linear encoding models (linearized regression) in order to predict the time-varying BOLD response of each voxel of each subject from the time varying stimulus. We have called this approach voxelwise modeling, VM for short. First, we constructed a baseline VM that used power spectrum and phoneme rate as regressorsto predict the BOLD response. We then subtracted this prediction from the measured BOLD response to obtain a response residual before fitting VMs based on phonemic and semantic identities. Subtracting the prediction from this baseline VM is needed in order to distinguish variance in the BOLD response that is simply due to the presence versus absence of phonemes or words from the variance that is dependent on the identity of phonemic and semantic features.

Second, four nested VMs were fitted to predict the BOLD response residual. A first order phonemic model was fitted to predict BOLD response residual from single phoneme features. The second order phonemic VM used both single phoneme and diphone features to predict the same response residual. The third order phonemic VM used single phonemes, diphones and triphones features as regressors. Finally, the phoneme-semantic model was obtained by using all three phonemic features with semantic features.

Before fitting the model weights (also known as model coefficients in the context of linear regression), the regressors X and the BOLD response Y were z-scored. BOLD response was z-scored separately for each story to control for the random effect of the story.

In order to account for the temporal integration time constants of both the neural processing and the BOLD response, our linear models predict the BOLD response at time t from signal features evaluated in four time windows of 2 seconds each and starting at t−2s, t−4s, t−6s and t−8s. This is accomplished by concatenating feature vectors that had been delayed by 2s, 4s, 6s, and 8s.

Due to the relatively large number of features relative to time points, we used regularized regression techniques to estimate the model weights to prevent overfitting. Regularization was achieved using Tikhonov regression with different levels of regularization used for different feature spaces and delays for each voxel and each subject.

Variance Partitioning

In order to quantify the variance and localize the cortical regions uniquely explained by individual feature spaces and any combination of feature spaces, we performed a variance partitioning procedure for We first fitted a joint phonemic model with all phonemic features (single phonemes, diphones and triphones). Then, we used the hyperparameters (to the optimal shrinkage) obtained from this joint phonemic model of each block of features in the banded regression so that the same sub space of features obtained by the ridge is used in all models. We computed the variance of seven possible partitions: the unique variance of each feature and all the pos- sible combinations of these features (single phonemes + diphones, diphones + triphones, single phonemes + triphone, single phonemes + diphones + tri- phones). Afterwards, we performed a variance correction on R2 that is used to eliminate biases in the R2 estimation that could lead to nonsensical results.
